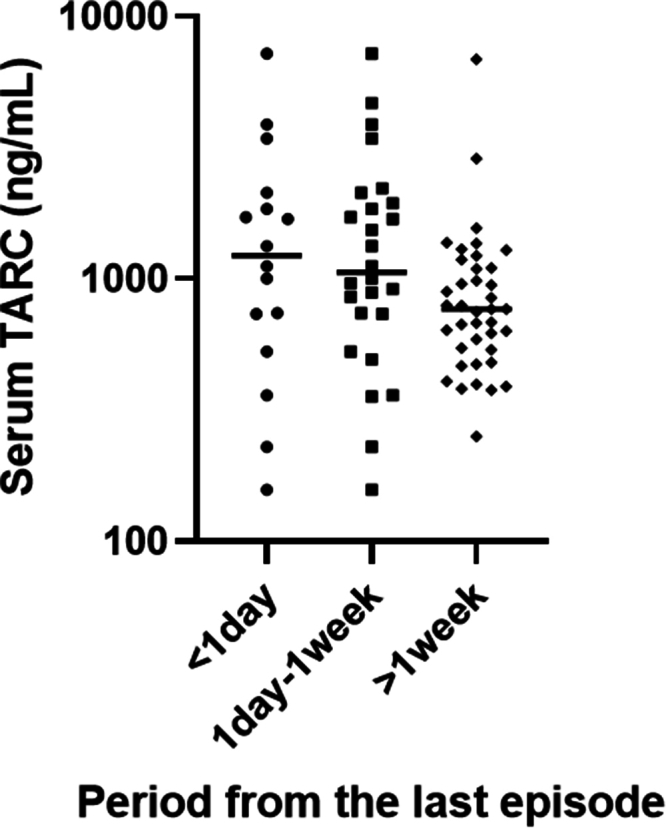# Diagnostic potential of allergen-specific lymphocyte stimulation test in hen’s egg yolk–induced, food protein–induced enterocolitis syndrome

**DOI:** 10.1016/j.jacig.2025.100596

**Published:** 2025-10-31

**Authors:** Shingo Yamada, Reiko Tokuda, Takahiro Nishida, Rei Kanai, Tomoyuki Arima, Takafumi Takase, Fumiko Iwai, Makiko Nakamoto, Mizuho Nagao, Keigo Kainuma, Tadashi Matsuda, Chiho Tatsumoto, Ryuji Yasui, Ryo Imakiire, Kana Hamada, Hisashi Nishimori, Kumiko Matsuo, Hiroyuki Hirai, Takao Fujisawa

**Affiliations:** a Institute for Clinical Research and Allergy Center, NHO Mie National Hospital, Tsu, Japan; b Tokuda Family Clinic, Ise, Japan; c Kainuma Clinic, Yokkaichi, Japan; d Matsuda Children’s Clinic, Kuwana, Japan; e Aozora Children’s Clinic, Kagoshima, Japan; f Yasui Kids and Allergy Clinic, Tokai, Japan; g Imakiire General Hospital, Kagoshima, Japan; h Hyogo Prefectural Kobe Children’s Hospital, Kobe, Japan; i Department of Pediatrics, Mie Prefectural General Medical Center, Yokkaichi, Japan; j Cell Biology Section, General Laboratory, BML Inc, Kawagoe, Japan

**Keywords:** Food protein–induced enterocolitis syndrome, diagnostic biomarkers, allergen-induced lymphocyte stimulation test, hen’s egg yolk, infant food allergies

## Abstract

**Background:**

Food protein–induced enterocolitis syndrome (FPIES) is a non–IgE-mediated food allergy frequently triggered by hen’s egg yolk (EY) in Japan. The lack of specific biomarkers hinders prompt diagnosis and appropriate management. Although oral food challenges are the diagnostic reference standard, their invasiveness and associated risks limit their feasibility in routine clinical practice.

**Objective:**

This study aimed to assess the diagnostic utility of the allergen-specific lymphocyte stimulation test (ALST) for EY-induced FPIES.

**Methods:**

We enrolled 71 infants diagnosed with EY-FPIES according to International Consensus Guidelines, along with 20 age-matched controls with allergic diseases other than FPIES. Peripheral blood mononuclear cells were stimulated with lipopolysaccharide-free EY and egg white (EW) extracts. The stimulation index (SI), representing antigen-specific lymphocyte responses, was calculated, and the EY/EW SI ratio was evaluated by receiver operating characteristic curve analysis.

**Results:**

Although EY-SI values were higher in the EY-FPIES group than in controls, the difference was not significant. In contrast, the EY-SI/EW-SI ratio showed modest diagnostic accuracy (area under curve = 0.68). At a cutoff of 1.75, sensitivity was 50.7%, specificity 80.0%, positive predictive value 90.0%, and negative predictive value 31.0%. Notably, EW-specific IgE correlated with the EY-SI/EW-SI ratio but not with EW-SI, suggesting that ALST was not due to simple cross-reactivity with EW.

**Conclusion:**

The EY/EW lymphocyte response ratio may serve as a supportive diagnostic biomarker for EY-FPIES. Despite its limited sensitivity, the high specificity and positive predictive value suggest that ALST could help reduce reliance on invasive oral food challenges in clinical practice.

Food protein–induced enterocolitis syndrome (FPIES) is a non–IgE-mediated food allergy characterized by delayed-onset gastrointestinal symptoms such as profuse vomiting, lethargy, and pallor.^1^ Unlike IgE-mediated allergies, FPIES does not involve immediate hypersensitivity reactions such as urticaria or anaphylaxis, making clinical diagnosis particularly challenging. Furthermore, FPIES commonly occurs in otherwise healthy infants without a history of atopic dermatitis, despite the well-established association between eczema and IgE-mediated food allergies.^2^ The absence of preceding atopic symptoms may contribute to delayed recognition of food allergy as an underlying cause, resulting in repeated episodes of vomiting before a correct diagnosis is made. These diagnostic delays impose considerable physical, emotional, and logistical burdens on affected children and their families.^3^

In Japan, hen’s egg yolk (EY) has become the most frequent trigger of FPIES.^4^^,^^5^ This trend may be partially attributable to national guidelines that recommend the early introduction of hen’s egg as a strategy to prevent IgE-mediated egg allergy.^6^ Although these guidelines do not specifically advocate for EY alone, traditional weaning practices in Japan typically introduce eggs in a stepwise manner, beginning with EY. As a result, early EY introduction has become common in practice. While early introduction of hen’s egg is anticipated to reduce the incidence of IgE-mediated egg allergy, its preventive effect has yet to be conclusively demonstrated.^7^ Nevertheless, the widespread adoption of early EY feeding may have inadvertently contributed to the rise in EY-FPIES cases.^8^ Given this emerging clinical pattern, further investigation is warranted to elucidate the immunopathology and to establish reliable diagnostic methods specific to EY-FPIES. These insights are also globally relevant, as early introduction of allergenic food such as peanut is being promoted in other countries as well, potentially leading to similar challenges in diagnosing and managing FPIES.^9^

Current diagnostic approaches for FPIES rely primarily on clinical observation and oral food challenges (OFCs), which are not only time-consuming but also pose a risk of severe adverse reactions.^10^ Several candidate biomarkers—such as thymus and activation-regulated chemokine (TARC),^11^ procalcitonin,^12^ neutrophil counts,^13^ and fecal proteins including hemoglobin, lactoferrin, and calprotectin^14^—have been proposed. However, their utility is limited by their transient elevation during acute episodes or in response to OFCs, thereby restricting their use in routine clinical practice. This highlights the need for noninvasive, reproducible biomarkers that can support diagnosis across both symptomatic and asymptomatic phases.

The allergen-specific lymphocyte stimulation test (ALST) has emerged as a potential diagnostic tool for non–IgE-mediated food allergies, including FPIES.^15^ By measuring lymphocyte proliferation in response to specific allergens, ALST may provide objective evidence of cell-mediated immune activation. Although promising results have been reported in cases of cow’s milk–induced FPIES,^16^ its utility in EY-FPIES remains largely unexplored.^17^ This study aimed to address this knowledge gap by evaluating the diagnostic performance of ALST in a well-characterized cohort of infants with EY-FPIES and appropriate controls, with the goal of identifying a reliable, immunologically relevant biomarker for clinical use.

## Methods

### Subjects

This multicenter study was conducted between February 1, 2022, and March 31, 2024, across 9 institutions in Japan. Participants included patients diagnosed with EY-FPIES and a control group. The diagnosis of EY-FPIES was established according to the International Consensus Guidelines for FPIES.^1^ The control group comprised children who were able to tolerate more than half an EY without any symptoms but had a diagnosis of IgE-mediated food allergy and/or atopic dermatitis.

Written informed consent was obtained from the parents or legal guardians of all participants. The study protocol was approved by the institutional review board of NHO Mie National Hospital (approval 2021-87).

### Data collection and laboratory measurements

Clinical background information, including demographic characteristics, medical history, and laboratory test results, was extracted from electronic medical records. Laboratory assessments included a complete blood count with differential, as well as measurements of serum TARC and total IgE levels. Allergen-specific IgE levels to EW, ovomucoid (OVM), and EY were measured with the ImmunoCAP system (Thermo Fisher Scientific, Uppsala, Sweden) following the manufacturer’s instructions.

### Antigen preparation

Antigens for the lymphocyte stimulation assays were prepared using a standardized protocol. Fresh hen’s eggs were externally disinfected with cotton pads soaked in 70% ethanol. A small incision was made in the eggshell, and a sterile syringe needle was carefully inserted into the yolk to aspirate the contents. The collected yolk was then freeze-dried, resuspended in phosphate-buffered saline, and adjusted to a final protein concentration of 100 mg/mL. The antigen preparation was stored at −80°C until use. EW antigens were prepared using an identical procedure, with a separate sterile needle inserted into the EW compartment to prevent cross-contamination.

To evaluate potential contamination of the EY fraction with EW proteins, specific EW allergens were quantified using commercial enzyme-linked immunosorbent assay (ELISA) kits: ovotransferrin (Immunology Consultants Laboratory, Portland, Ore), ovalbumin (Institute of Tokyo Environmental Allergy, Tokyo, Japan), and OVM (BioFront Technologies, Tallahassee, Fla). Quantitative ELISA confirmed only trace levels of contamination in the EY preparation: ovotransferrin (0.11%), ovalbumin (0.35%), and OVM (0.09%). These levels were well below the thresholds known to induce lymphocyte responses in IgE-mediated egg allergy. Although the preparation procedure and validation steps were designed to minimize contamination, potential immunologic effects from residual EW proteins could not be entirely excluded. This consideration prompted the use of an EY/EW stimulation index ratio in the subsequent analysis to account for any influence from EW-derived antigens.

Given the potential presence of lipopolysaccharides (LPS) in protein preparations and their ability to induce nonspecific lymphocyte activation—thereby confounding the results of allergen-induced lymphocyte stimulation assays^18^—LPS concentrations in the EY and EW extracts were measured using a kinetic turbidimetric limulus amebocyte lysate assay (Wako Chemicals, Tokyo, Japan). All antigen preparations contained LPS levels below the detection limit of 0.1 pg/mL

### Allergen-induced lymphocyte stimulation test (ALST)

Peripheral blood mononuclear cells were isolated and suspended at a concentration of 1 × 10^6^ cells/mL in RPMI 1640 medium (Kojin Bio, Saitama, Japan) supplemented with 10% human AB serum (Golden West Diagnostics, Temecula, Calif). The cells were cultured in the presence of EY and EW extracts at concentrations of 1, 11, 33, 100, and 300 μg/mL for 6 days. Lymphocyte proliferation was assessed by the incorporation of tritiated thymidine (Revvity, Waltham, Mass), and radioactivity was measured with a liquid scintillation counter. Results were expressed as a stimulation index (SI), defined as the ratio of counts per minute in antigen-stimulated wells to those in unstimulated control wells. This method enabled the quantification of antigen-specific lymphocyte responses to EY and EW.

### Modified SI

Although quantitative ELISA confirmed only trace levels of EW protein contamination in the EY antigen preparation, the presence of ovotransferrin, ovalbumin, and OVM could not be entirely excluded as potential contributors to lymphocyte activation. To account for possible nonspecific effects, we calculated a modified SI (mSI), defined as the ratio of EY-SI to EW-SI. This index was intended to reflect the relative selectivity of lymphocyte responses to EY antigens, normalized to those elicited by EW antigens, and to mitigate the potential influence of residual EW proteins on assay results.

### Statistical analysis

Categorical variables were analyzed by Fisher exact test. For ordinal categorical variables, the Mantel-Haenszel chi-square test for trend was applied to account for the ordered nature of the data. Continuous variables were compared by the Mann-Whitney *U* test after confirming nonnormal distribution by the Shapiro-Wilk test.

The diagnostic performance of ALST was evaluated by receiver operating characteristic (ROC) curve analysis. The area under the curve (AUC) was calculated to assess discriminatory ability. Sensitivity, specificity, positive predictive value (PPV), and negative predictive value (NPV) were also calculated to characterize diagnostic accuracy. Associations between two variables were assessed by the Spearman rank correlation analysis.

Two-tailed *P* < .05 was considered statistically significant. All statistical analyses and data visualizations were performed by GraphPad Prism v10 software (GraphPad Software, La Jolla, Calif).

## Results

### Study participants

A total of 96 children were initially recruited from 9 participating centers on the basis of clinical suspicion of EY-FPIES, in accordance with the International Consensus Guidelines.^1^ After enrollment, expert review was conducted to rigorously reapply the diagnostic criteria defined in the guidelines using all available clinical information.

**Fig 1:**
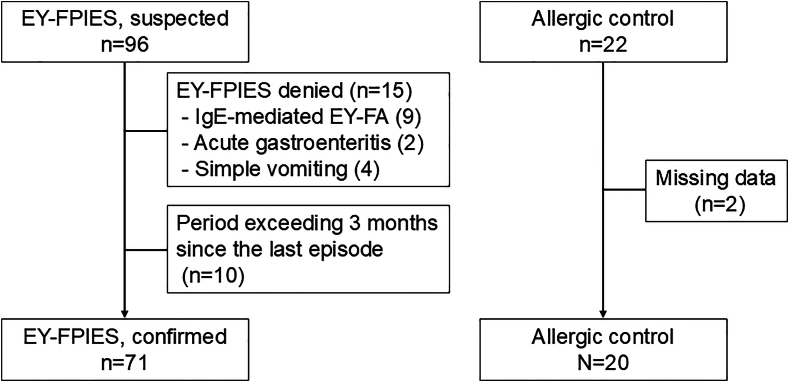
Participant recruitment flowchart. Ninety-six children were initially enrolled onto EY-FPIES group. Of these, 25 were excluded for specific reasons shown here, resulting in 71 participants in final EY-FPIES group. Control group initially comprised 22 children, of whom 2 were excluded because of incomplete data, yielding 20 participants in final control group.

Nine children were excluded after being diagnosed with IgE-mediated EW allergy, which was based on elevated EW-specific IgE levels and symptoms occurring exclusively after EW ingestion. Two children were excluded after a diagnosis of infectious gastroenteritis, as similar gastrointestinal symptoms developed in multiple family members after enrollment. Four children were classified as having nonspecific vomiting, defined by frequent vomiting triggered by various weaning foods, without reproducible symptoms on EY ingestion or other clinical features supporting a diagnosis of FPIES. These classifications were confirmed through consensus by at least two board-certified pediatric allergists. In addition, 10 children were excluded because more than 3 months had passed between their most recent FPIES-like episode and the time of blood sampling, suggesting possible spontaneous resolution of the disease. Consequently, 71 children were included in the final EY-FPIES group. The control group initially comprised 22 children, of whom 2 were excluded because of incomplete data, resulting in 20 participants with data available for analysis (Fig 1).

### Clinical characteristics of participants

The clinical characteristics of the EY-FPIES and control groups are summarized in Table I. The median age of the control group was approximately 4 months higher than that of the EY-FPIES group; this difference was statistically significant. This age gap likely reflects the typical clinical course of FPIES, which often presents shortly after the introduction of complementary foods, prompting diagnostic evaluations around 8 months of age. In contrast, children in the control group—diagnosed with other allergic conditions—rarely undergo blood sampling at such an early stage because of the absence of immediate clinical indications as well as because of ethical considerations. The timing of EY introduction did not differ significantly between groups. In EY-FPIES patients, the median interval from EY introduction to symptom onset was 2 months.

**Table I:** Participant characteristics

Characteristic	EY-FPIES (n = 71)	Control (n = 20)	*P* value
Male sex, no. (%)	31 (43)	12 (60)	.2114
Age (months), median (IQR)	8 (8-10)	12 (9-16)	.0017
Age at EY introduction (months), median (IQR)	6 (6-7)	7 (6-8)	.1017
Interval (months) from EY introduction to FPIES onset, median (IQR)	2 (1-3)	—	—
Perinatal history			
Gestational weeks, median (IQR)	39.1 (38.3-40.0)	39.6 (38.5-40.1)	.2755
Birth weight (g), median (IQR)	3040 (2843-3284)	3101 (2801-3551)	.6283
Caesarean section, no. (%)	15 (21)	4 (20)	>.9999
Antibiotics receipt at <28 days of age, no. (%)	3 (4)	1 (5)	>.9999
No. undergoing surgery at <28 days of age	0	0	—
Cow’s milk formula receipt, no. (%)			.8804
Never	0	1 (5)	
At maternity hospital	9 (13)	2 (10)	
Only few times	15 (22)	3 (15)	
Regularly	46 (67)	14 (70)	
Not reported	2	0	
Comorbid allergy, no. (%)			
Infant eczema or atopic dermatitis	37 (66)	17 (85)	.1648
IgE-mediated food allergy (including hen’s egg allergy)∗	18 (25)	12 (60)	.0063
Wheezing	1 (1)	1 (5)	.3932
Family history/environment, no. (%)			
Siblings	18 (30)	12 (60)	.0178
Family history of allergy	43 (61)	15 (75)	.2979
Passive smoking	8 (11)	3 (15)	.7010
Pets	11 (15)	6 (70)	.1922

*IQR,* Interquartile range.

∗ In EY-FPIES group, IgE-mediated food allergy was based on sensitization without history of ingestion, including peanut (n = 16), wheat (n = 1), and soy (n = 1). OFCs had not been performed because of young age and prioritization of FPIES management. In control group, all 12 cases of IgE-mediated food allergy were confirmed egg allergy, diagnosed via OFC with controlled partial egg intake.

The prevalence of IgE-mediated food allergies, including hen’s egg allergy, was significantly higher in the control group than in the EY-FPIES group. Additionally, the proportion of participants with siblings was greater in the control group, which may influence the development or presentation of allergic diseases. However, no significant differences were observed between the two groups in terms of perinatal factors, formula feeding history, or family history of allergic disease—factors that have been proposed as potential risk factors for FPIES.^19^

### Laboratory findings

The distribution of hen’s egg protein–specific IgE antibody titers by class is shown in Table II. The control group exhibited a higher proportion of participants with elevated titers compared to the EY-FPIES group. For both EW-specific IgE and OVM-specific IgE, the chi-square test for trend demonstrated statistically significant differences between groups. Although the distribution of EY-specific IgE titers was skewed and the difference did not reach statistical significance, the control group still showed a higher proportion of elevated values. No statistically significant differences were observed between the groups in other laboratory parameters, including white blood cell counts, eosinophils, neutrophils, lymphocytes, or serum TARC levels. In addition, analysis of TARC levels stratified by time since the last FPIES episode in the EY-FPIES group revealed no significant differences (see Fig E1 in this article’s Online Repository available at www.jaci-global.org).

**Table II:** Participants’ laboratory data

Characteristic	Variable	EY-FPIES (n = 71)	Control (n = 20)	*P* value
EW-specific IgE	Class 0, no. (%)	33 (46.5)	2 (10.0)	.0001∗
	Class 1, no. (%)	13 (18.3)	3 (15.0)	
	Class 2, no. (%)	14 (19.7)	6 (30.0)	
	Class 3, no. (%)	10 (14.1)	5 (25.0)	
	Class 4+, no. (%)	1 (1.4)	4 (20.0)	
OVM-specific IgE	Class 0, no. (%)	58 (81.7)	9 (45.0)	.0034∗
	Class 1, no. (%)	4 (5.6)	4 (20.0)	
	Class 2, no. (%)	7 (9.9)	4 (20.0)	
	Class 3, no. (%)	1 (1.4)	3 (15.0)	
	Class 4+, no. (%)	1 (1.4)	0	
EY-specific IgE	Class 0, no. (%)	47 (66.2)	6 (31.6)	—
	Class 1, no. (%)	0	3 (15.8)	
	Class 2, no. (%)	24 (33.8)	7 (36.8)	
	Class 3, no. (%)	0	3 (15.8)	
	Class 4+, no. (%)	0	0	
WBC (/μL)	Median (IQR)	10,835 (9173-12,110)	8770 (8020-11,930)	.1041†
Eosinophils (/μL)	Median (IQR)	222 (142-409)	329 (178-453)	.2340†
Neutrophils (/μL)	Median (IQR)	2396 (1571-3735)	2363 (1633-3204)	.6089†
Lymphocytes (/μL)	Median (IQR)	7041 (5732-7759)	5805 (4635-8111)	.2263†
Total IgE (IU/mL)	Median (IQR)	13 (5-28)	54 (21-137)	<.0001†
Serum TARC (pg/mL)	Median (IQR)	853 (540-1346)	604 (373-1222)	.2089†

*IQR,* Interquartile range; *WBC,* white blood cell count.

∗ Chi-square for trend.

† Mann-Whitney *U* test.

### Symptoms of EY-FPIES

Symptoms associated with EY-FPIES are summarized in Table III. Vomiting was reported in all cases, with a median (range) onset time of 2.5 (1-4) hours after ingestion and a recovery time of 4 (1-12) hours. Other frequently observed symptoms included decreased activity (92%) and pallor (75%). In addition, 7% of the patients required intravenous fluid administration, and 39% required hospitalization. These clinical features indicate that the enrolled EY-FPIES patients had disease of sufficient severity to justify their inclusion in the study cohort, reflecting the typical symptomatic presentation of this condition.

**Table III:** Symptoms of EY-FPIES

Symptom	Overall (N = 71)	Severity∗		*P* value
		Mild (n = 33)	Moderate to severe (n = 37)	
Vomiting, no. (%)	71 (100)			
No. of repetitions, median (range)	4 (1-7)	2 (1-2)	5 (3-7)	—†
Hours from ingestion to onset, median (range)	2.5 (1-4)	2.3 (1-4)	2.8 (1-4)	.497
Hours to recovery, median (range)	4 (1-12)	3 (1-12)	5 (1-12)	.048
Decreased activity level, no. (%)	65 (92)	29 (78)	34 (92)	.699
Pallor, no. (%)	53 (75)	24 (65)	28 (76)	.392
Intravenous hydration, no. (%)	5 (7)	0	4 (11)	—†
Diarrhea, no. (%)	10 (14)	8 (22)	5 (14)	.357
Hypotension, no. (%)	0	0	0	—‡
Hypothermia, no. (%)	0	0	0	—‡
Hospitalization, no. (%)	28 (39)	0	28 (76)	—†

Median (IQR) intake of boiled EY at time of symptom elicitation was 5 (5-7.5) g, or approximately ¼ of whole EY. Corresponding EY protein intake was estimated at median (IQR) 805 (805-1208) mg, calculated using protein content of 16.1 g per 100 g of edible portion, as calculated by the *Standard Tables of Food Composition in Japan* (Tokyo: Ministry of Education, Culture, Sports, Science and Technology; 2020, 8th ed., 2023 supplement). Among nondefining variables, only recovery time differed significantly (longer in moderate-to-severe group); all others were not significant. Group comparisons used chi-square/Fisher exact test or Mann-Whitney *U* test, as appropriate.

*IQR,* Interquartile range.

∗ Severity operational definition was based on 2017 International Consensus Guidelines,^1^ as follows: mild, ≤2 vomiting episodes within 1 to 4 hours after ingestion; and moderate to severe, ≥3 vomiting episodes or any intravenous hydration, hypotension, hypothermia, or hospitalization.

† Severity-defining variable; therefore, between-group testing was not performed.

‡ No events were observed; variables were thus omitted from hypothesis testing.

### Antigen-specific lymphocyte stimulation test (ALST)

**Fig 2:**
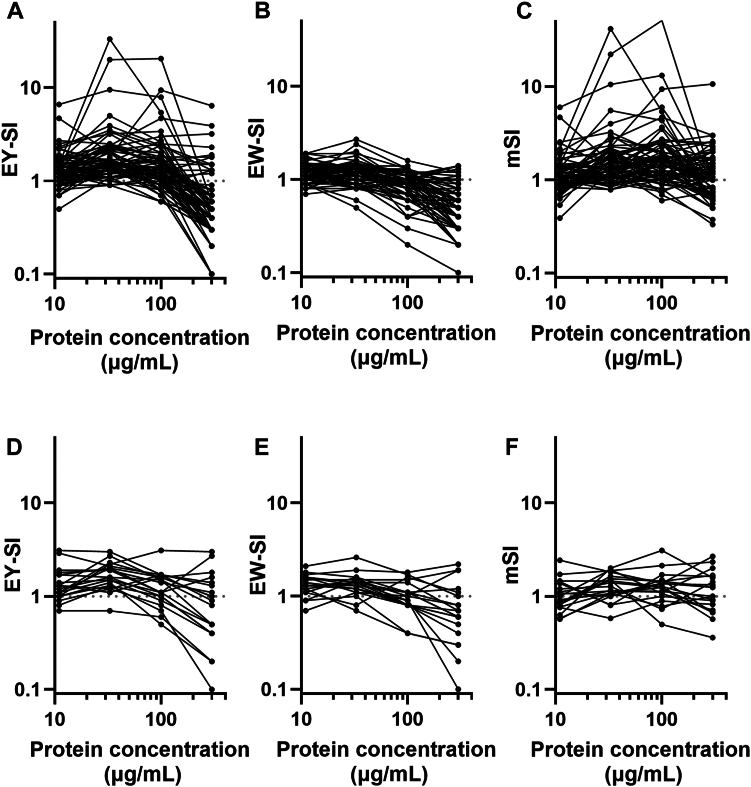
Lymphocyte responses to EY and EW extracts. Lymphocyte SI data are presented for EY-FPIES group **(A-C)** and allergic control group **(D-F)**. SI values are presented for EY (*A* and *D*) and EW (*B* and *E*), along with mSI , defined as ratio of EY-SI to EW-SI (*C* and *F*). Each *line* represents SI or mSI response of individual participant across varying concentrations of EY and EW antigens; *red dashed lines,* threshold of SI = 1. Allergic control group comprises children able to tolerate more than ½ EY without symptoms but had diagnosis of IgE-mediated food allergy and/or atopic dermatitis.

Lymphocyte SI responses to EY and EW were assessed across all subjects (Fig 2). In the EY-FPIES group, lymphocyte responses to EY (SI > 1) were observed in the majority of cases (Fig 2, *A*), indicating cellular activation in response to EY antigens. In contrast, responses to EW were generally lower in this group; however, a subset of participants also exhibited SI values greater than 1 for EW (Fig 2, *B*).

### mSI

Given previous reports indicating that specific IgE levels may correlate with ALST responses to EW,^20^ we hypothesized that EY-FPIES patients sensitized to EW might exhibit lymphocyte reactivity to trace amounts of EW proteins present in the EY antigen preparation. To address this potential confounding effect, a mSI was calculated as the ratio of EY-SI to EW-SI, as described above in the Methods section. Analysis of mSI further emphasized the differential reactivity between EY and EW. In the EY-FPIES group, a marked predominance of lymphocyte activation to EY over EW was observed (Fig 2, *C*), supporting the antigen-specific nature of the cellular response. In contrast, most participants in the control group showed low SI values for both EY and EW (Fig 2, *D* and *E*), and correspondingly low mSI values (Fig 2, *F*).

### Diagnostic performance and clinical significance of ALST

Variability in the antigen concentration at which the peak SI occurred was observed among individual patients, with most exhibiting peak responses at either 33 μg/mL or 100 μg/mL. This variability likely reflects individual differences in lymphocyte reactivity. To better capture each subject’s specific immune response and to ensure consistency in interindividual comparisons, SI values were calculated at both 33 μg/mL and 100 μg/mL, and the higher of the two was designated as the representative SI, or peak SI, for each antigen.

**Fig 3:**
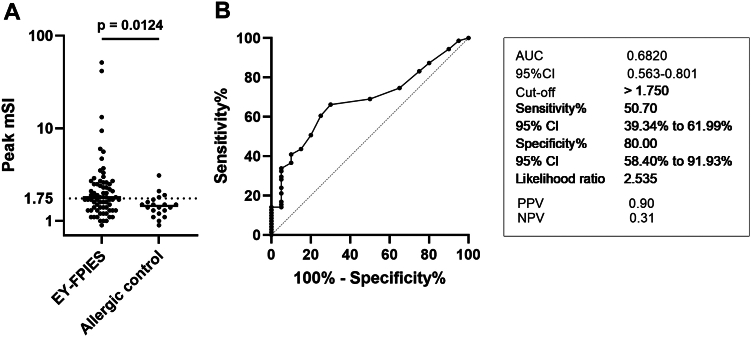
ROC analysis of mSI. **(A)** Distribution of peak mSI values in EY-FPIES and allergic control groups. **(B)** ROC curve for peak mSI, with AUC of 0.68 (95% confidence interval, 0.56-0.80). Using optimal cutoff value of 1.75, sensitivity was 50.7%, specificity was 80.0%, PPV was 90.0%, and NPV was 31.0%. Allergic control group comprises children able to tolerate more than ½ EY without symptoms but had diagnosis of IgE-mediated food allergy and/or atopic dermatitis.

The peak mSI, calculated as the ratio of EY-SI to EW-SI, was significantly higher in the EY-FPIES group compared to the control group (Fig 3, *A*). ROC curve analysis yielded an AUC of 0.68 (95% confidence interval, 0.56-0.80; Fig 3, *B*). At an optimal cutoff value of 1.75, determined by the Youden index, the sensitivity was 50.7% and specificity was 80.0%, with a PPV of 90.0% and an NPV of 31.0%.

**Fig 4:**
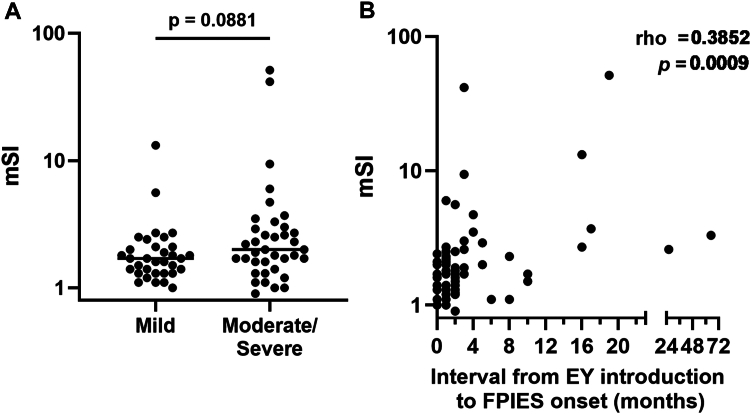
Association of mSI with clinical severity and latency to symptom onset in EY-FPIES patients. **(A)** Comparison of mSI between patients with mild vs moderate-to-severe symptoms by Mann-Whitney *U* test. **(B)** Correlation between mSI and interval from EY introduction to FPIES onset assessed by Spearman rank correlation. *Rho* indicates Spearman rank correlation coefficient (ρ).

To explore the potential clinical relevance of interindividual variability in mSI values, we performed a *post hoc* analysis comparing mSI values between patients classified as having mild or moderate-to-severe symptoms.^1^ Although the difference did not reach statistical significance, mSI tended to be higher in the moderate-to-severe group (Fig 4, *A*).

Next, to assess whether the duration of antigen exposure might influence immune activation, we examined the relationship between mSI and the interval from the introduction of EY to symptom onset. A significant positive correlation was observed, indicating that longer latency may be associated with more robust T cell reactivity (Fig 4, *B*).

### Correlation of EW-specific IgE with ALST responses

**Fig 5:**
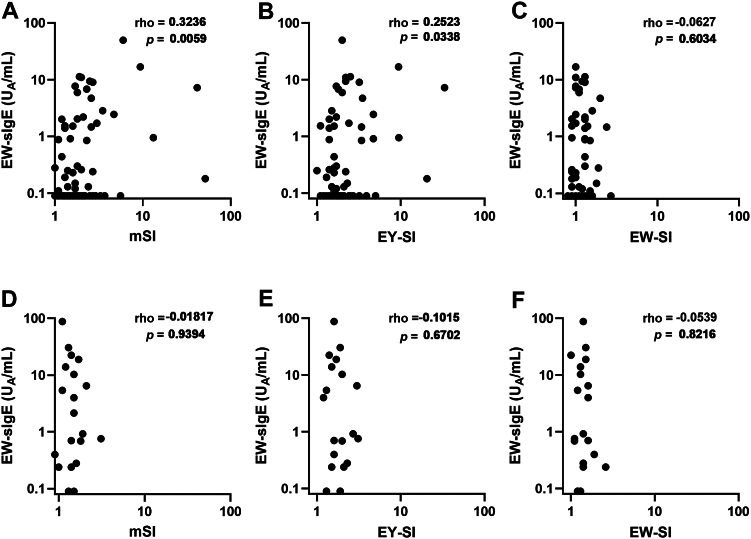
Scatterplots showing correlations between EW-sIgE levels and ALST parameters in EY-FPIES and control groups. Scatterplots compare EW-sIgE levels (y-axis, kU_A_/L) with mSI, EY-SI, or EW-SI (x-axis). **(A-C)** Patients with EY-FPIES. **(D-F)** Control subjects. Spearman rank correlation coefficient *(r)* and associated *P* values are shown. *Bold* values indicate statistically significant correlations (*P* < .05).

To examine whether the lymphocyte response to EY antigens was influenced by sensitization to EW, we performed correlation analyses between EW-specific IgE (sIgE) and ALST-derived stimulation indices in both the FPIES and control groups (Fig 5). In the FPIES group, a significant positive correlation was observed between EW-sIgE and mSI, as well as a weaker but significant correlation with EY-SI. In contrast, no correlation was found between EW-sIgE and EW-SI. In the control group, EW-sIgE levels varied widely, but they showed no significant correlation with mSI, EY-SI, or EW-SI.

## Discussion

This study demonstrates that ALST holds potential as a diagnostic tool for EY-FPIES. In the absence of identified FPIES-specific allergenic components, diagnostic specificity was enhanced through the use of the mSI, which was designed to control for the potential confounding effects of residual EW proteins. This adjustment was particularly important given that crude antigen extracts were used in the assay. In addition, the careful preparation of antigen extracts to exclude LPS likely minimized nonspecific immune activation, thereby improving assay reliability. Taken together, these results suggest that ALST, when interpreted using the mSI, may represent a safer and less invasive alternative to OFC, which remains the reference standard but is associated with significant risk and logistical burden.^21^^,^^22^

Previous studies have investigated the utility of ALST in the diagnosis of hen’s egg–induced FPIES.^17^^,^^23^^,^^24^ While these studies have provided valuable insights, several methodologic limitations should be acknowledged. First, most were conducted at a single center and included relatively small sample sizes—typically fewer than 35 cases—potentially limiting statistical power and generalizability. Second, the methods for antigen preparation were not standardized across studies, which may have introduced variability in lymphocyte responses and hindered reproducibility. Third, comparison groups in these studies often consisted of subjects stratified by OFC outcomes alone. Although such an approach facilitates internal contrast, it may not fully represent an appropriate comparison group for evaluating diagnostic specificity in broader clinical settings.

To address the limitations identified in previous studies, we incorporated several methodologic refinements into the present study. First, we conducted a multicenter investigation to increase sample size and capture a broader range of clinical settings, thereby enhancing both the generalizability and statistical robustness of our findings. Second, we defined a control group consisting of children with other clinically relevant allergic conditions, which allowed for more precise differentiation between EY-FPIES and other forms of food allergy. Third, in contrast to earlier studies lacking standardized antigen preparation protocols, we developed a carefully controlled procedure for preparing crude egg antigens. EY extracts were validated for protein purity using 3 separate ELISA assays and were confirmed to contain less than 0.4% EW proteins. In addition, LPS concentrations were below the detection limit (<0.1 pg/mL), minimizing the risk of nonspecific lymphocyte activation due to endotoxin contamination.^18^ Although the contamination with EW proteins was minimal, the potential influence of residual EW allergens could not be completely excluded given the use of crude extracts. To address this inherent limitation, we introduced a mSI, designed to adjust for background reactivity and enhance the specificity of lymphocyte responses to EY antigens. While these refinements do not eliminate all challenges associated with ALST, they represent meaningful advances toward improving the diagnostic precision and applicability of this test in the context of EY-FPIES.

To further assess antigen specificity, we analyzed the relationship between EW-sIgE– and ALST-derived indices. In the FPIES group, EW-sIgE was positively correlated with mSI but not with EW-SI, suggesting that the observed lymphocyte responses are not merely due to cross-reactivity with EW but may reflect a heightened state of immune activation. Although the clinical significance of this activation remains unclear and its association with long-term outcomes^25^^,^^26^ requires further investigation, our findings indicate that EW-sIgE–positive patients tend to show stronger cellular responses to EY antigens.

The immunologic mechanisms and routes of sensitization in FPIES remain unclear.^27^ Of note, in our cohort, symptoms did not occur immediately after EY introduction but after a delay, and a longer interval was modestly associated with higher mSI values. This suggests that repeated low-dose exposure through the gastrointestinal tract may lead to immune priming. The gradual introduction of EY during complementary feeding in Japan may contribute to such priming in susceptible infants.

This study has several limitations that warrant consideration. First, the diagnosis of FPIES was not universally confirmed by OFC, which remains the reference standard for FPIES diagnosis. However, the diagnostic framework applied in this study was based on the internationally accepted 2017 consensus guidelines,^1^ which have been robustly validated. A recent study suggested that patients not fulfilling these criteria often present with milder symptoms and may instead reflect features of IgE-mediated food allergy.^28^ Therefore, the inclusion criteria are likely enriched for more definitive, non–IgE-mediated FPIES cases, potentially enhancing the specificity of the findings.

Second, while mSI demonstrated high specificity (80%) and PPV (90%), its sensitivity (50.7%) and NPV (31%) were limited. Nonetheless, a positive mSI strongly supports an FPIES diagnosis and may help avoid invasive OFCs. Notably, mSI was numerically higher in moderate-to-severe cases in exploratory analyses, but differences were not statistically significant; this trend may indicate greater risk and could support deferring OFC.

Third, the small control group limits statistical power and may have led to an overestimation of the PPV. Although controls were carefully selected to reflect allergic backgrounds similar to the FPIES group, recruiting more participants was not feasible as a result of ethical and practical constraints in obtaining blood from healthy infants. Still, the findings are encouraging and warrant validation in larger studies.

Fourth, this study was conducted in Japan, where early EY introduction is common and EY-FPIES is more prevalent. This cultural and dietary context may limit the generalizability of our findings. FPIES triggers vary internationally,^29^ so future multicenter studies including diverse populations and food allergens will be needed to validate the utility of ALST in broader clinical settings.

In conclusion, this study demonstrates the potential of ALST and its mSI to improve diagnostic specificity for EY-FPIES, even with crude antigens. While not a substitute for OFC because of its limited sensitivity, ALST’s high specificity and noninvasiveness support its role as a complementary tool, especially in high-probability cases. These findings lay the groundwork for future studies to validate and expand its clinical application.

**Key messages**

• ALST demonstrated relatively high specificity and PPV for diagnosing EY-FPIES, not only during acute phases but also in asymptomatic periods.
• Unlike other biomarkers that assess inflammatory responses, ALST evaluates antigen reactivity, enabling its use even in symptom-free periods.

## Declaration of generative AI and AI-assisted technologies in the writing process

The authors used ChatGPT (OpenAI) to assist in language editing. All scientific content was developed and verified by the authors.

## Disclosure statement

Partially supported by the Nipponham Foundation for the Future of Food, Japan. The cost of ALST measurements was covered by BML Inc. The company had no role in the study design, data analysis, or preparation of the report.

Disclosure of potential conflict of interest: T.F. served as a consultant for BML Inc and has received lecture fees from Maruho, Sanofi, GSK, and Torii Pharmaceutical. M.N. has received lecture fees from Maruho, AbbVie, Otsuka Pharmaceutical, Sanofi, Torii Pharmaceutical, and Eli Lilly. K.M. and H.H. are employees of BML Inc, which provided funding for this research. The rest of the authors declare that they have no relevant conflicts of interest.

## Figures and Tables

**Supplementary Figure:**